# Chemistry of Organophosphonate Scale Growth lnhibitors: 3. Physicochemical Aspects of 2-Phosphonobutane-1,2,4-Tricarboxylate (PBTC) And Its Effect on CaCO_3_ Crystal Growth

**DOI:** 10.1155/BCA.2005.135

**Published:** 2005

**Authors:** Konstantinos D. Demadis, Panos Lykoudis

**Affiliations:** Department of Chemistry University of Crete 300 Leoforos Knossos Crete Heraklion GR-71409 Greece

**Keywords:** phosphonates, carboxylates, PBTC, calcium carbonate, crystal modifiers, inhibition, biocides

## Abstract

Industrial water systems often suffer from undesirable inorganic deposits, such as calcium carbonate, calcium phosphates, calcium sulfate, magnesium silicate, and others. Synthetic water additives, such as phosphonates and phosphonocarboxylates, are the most important and widely utilized scale inhibitors in a plethora of industrial applications including cooling water, geothermal drilling, desalination, etc. The design of efficient and cost-effective inhibitors, as well as the study of their structure and function at the molecular level are important areas of research. This study reports various physicochemical aspects of the chemistry of PBTC (PBTC = 2-phosphonobutane-1,2,4-tricarboxylic acid), one of the most widely used scale inhibitors in the cooling water treatment industry. These aspects include its CaCO_3_ crystal growth inhibition and modification properties under severe conditions of high CaCO_3_ supersaturation, stability towards oxidizing microbiocides and tolerance towards precipitation with Ca^2+^. Results show that 15 ppm of PBTC can inhibit the formation of by ∼35 %, 30 ppm by ∼40 %, and 60 ppm by ∼44 %. PBTC is virtually stable to the effects of a variety of oxidizing microbiocides, including chlorine, bromine and others. PBTC shows excellent tolerance towards precipitation as its Ca salt. Precipitation in a 1000 ppm Ca^2+^ (as CaCO_3_) occurs after 185 ppm PBTC are present.


### Chemistry of Organophosphonate Scale Growth Inhibitors: 3. Physicochemical Aspects of 2-Phosphonobutane-1,2,4-Tricarboxylate (PBTC) And Its Effect on $CaCO_3$ Crystal Growth §

Konstantinos D. Demadis* and Panos Lykoudis

*Department of Chemistry, University of Crete,*
*300 Leoforos Knossos, Heraklion, Crete, Greece GR-71409*

### GRAPHICAL ABSTRACT

This study reports various physicochemical aspects of the chemistry of PBTC that include inhibition of $CaCO_3$ crystal growth and modification properties under severe conditions of high $CaCO_3$ supersaturation, stability towards oxidizing microbiocides and tolerance towards precipitation with $Ca^{2+}$.

**CaCO3 crystals (Untreated)**

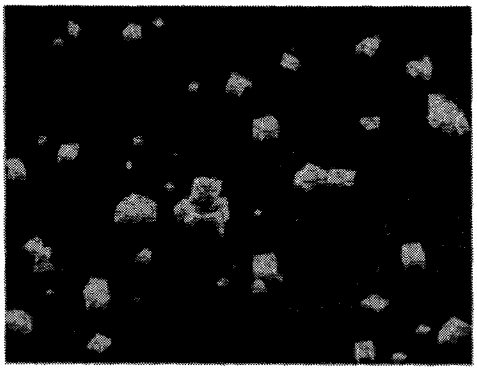

**Bar = 20 μ**

**CaCO3 crystals (treated with PBTC)**

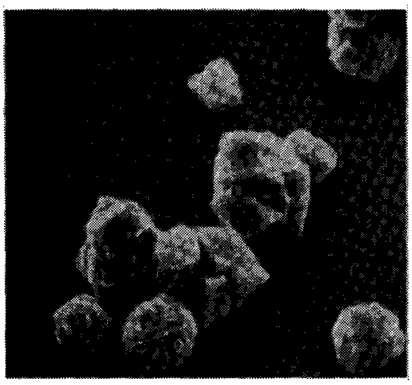

**Bar = 10 μ**

### ABSTRACT

Industrial water systems often suffer from undesirable inorganic deposits, such as calcium carbonate, calcium phosphates, calcium sulfate, magnesium silicate, and others. Synthetic water additives, such as phosphonates and phosphonocarboxylates, are the most important and widely utilized scale inhibitors in a

§ Part 2, See preceding paper.

* Phone: +30 2810 393651, fax: +30 2810 393601, e-mail: demadis@chemistry.uoc.gr plethora of industrial applications including cooling water, geothermal drilling, desalination, etc. The design of efficient and cost-effective inhibitors, as well as the study of their structure and function at the molecular level are important areas of research. This study reports various physicochemical aspects of the chemistry of PBTC (PBTC = 2-phosphonobutane-1,2,4-tricarboxylic acid), one of the most widely used scale inhibitors in the cooling water treatment industry. These aspects include its $CaCO_3$ crystal growth inhibition and modification properties under severe conditions of high $CaCO_3$ supersaturation, stability towards oxidizing microbiocides and tolerance towards precipitation with $Ca^{2+}$. Results show that 15 ppm of PBTC can inhibit the formation of by ~35 %, 30 ppm by ~40 %, and 60 ppm by ~44 %. PBTC is virtually stable to the effects of a variety of oxidizing microbiocides, including chlorine, bromine and others. PBTC shows excellent tolerance towards precipitation as its Ca salt. Precipitation in a 1000 ppm $Ca^{2+}$ (as $CaCO_3$) occurs after 185 ppm PBTC are present.

**Keywords:** phosphonates, carboxylates, PBTC, calcium carbonate, crystal modifiers, inhibition, biocides

### Glossary

| | |
|---|---|
| **HEDP** | hydroxyethylidenephosphonic acid |
| **AMP** | amino-methylene-tris-phosphonic acid |
| **PBTC** | Phosphonobutane-1,2,4-Tricarboxylic acid |
| **BCDM** | 1-bromo-3-chloro-5,5-dimethyl-hydantoin |
| **Calcium tolerance** | ability of an inhibitor to remain soluble in the presence of $Ca^{2+}$ |
| **Cycles of concentration** | Concentration increase of ions as compared to their initial concentration in the raw water |
| **Dispersancy** | Prevention of scale deposition on a surface |
| **Blowdown** | Designed loss of process cooling water and its replacement with "fresh" water in order to maintain a certain pre-specified level of conductivity |
| **Biocides** | Water additives that control microbiological growth. They can be either oxidizing or non-oxidizing. |
| **"Bromine"** | Term in the water treatment sector that is associated with $BrO^-$, not $Br_2$ |
| **"Chlorine"** | Term in the water treatment sector that is associated with $ClO^-$, not $Cl_2$ |
| **Stabilized Halogens** | $XO^-$ (X = halogen) with additives that render them less aggressive |

### INTRODUCTION

Calcium carbonate /1/ and calcium phosphate(s) /2/ are the most frequently encountered deposits in industrial water systems. Their accumulation greatly diminishes effective heat transfer, interferes with fluid flow, facilitates corrosion processes, and can worsen microbiological fouling /3/. These phenomena are most critical in cooling water applications, where incoming water passes through a heat exchanger, cools a "hot" process and is sent back to repeat the same cooling process after it is cooled by forced evaporation /4/. This water loss by evaporative cooling results in high supersaturation levels of dissolved ions. Eventually, massive precipitation of sparingly soluble mineral salts can occur, either in bulk or on a surface that, in some cases, causes catastrophic operational failures. These usually require chemical and/or mechanical cleaning of the adhered scale, in the aftermath of a scaling event. Silica and silicate salts are such examples /5/.

Scale growth can be mitigated by use of scale inhibitors. They are key components of any chemical water treatment added to process waters in "ppm" quantities and usually work synergistically with dispersant polymers /6,7/.

Phosphonates belong to a fundamental class of such compounds /8/ used extensively in cooling water treatment programs /9/, oilfield applications /10/ and corrosion control /11/. PBTC, HEDP (hydroxy-ethylidenediphosphonate) and AMP (amino-*tris*-methylenephosphonate) are "popular" and effective commercial scale inhibitors (Figure 1) /12/. Phosphonates are thought to achieve scale inhibition by adsorbing onto specific crystallographic planes of a growing crystal nucleus after a nucleation event. This adsorption prevents further crystal growth and agglomeration into larger aggregates /13/.

Study of phosphonates is attracting additional interest due to their potential uses in sequestering toxic metal ions in industrial effluents. Moreover, their established use as bone resorption agents and in treatments for osteoporosis makes them desirable from a biological/pharmaceutical perspective.

Understanding the function of scale inhibitors requires a closer look at the molecular level of their possible function. The present study aims toward this direction and reports the inhibition properties of 2-phosphonobutane-1,2,4-tricarboxylic towards $CaCO_3$ crystal growth inhibition under high supersaturation conditions, as well as its stability towards oxidizing biocides and $Ca^{2+}$ precipitation.

### EXPERIMENTAL SECTION

### Preparations

All phosphonates were obtained from Solutia UK (Newport, United Kingdom). PBTC is available in acid form under the commercial name Dequest 7000 as 50 % w/w solution in water and was used as received. Aqueous solutions of PBTC are infinitely stable if common preservation practices are applied.

Stock solutions were prepared in deionized water as follows: $CaCl_2{\cdot}2H_2O$ 10,000 ppm (as $CaCO_3$); $CaCl_2{\cdot}2H_2O$ 20,000 ppm (as $CaCO_3$); $MgSO_4{\cdot}7H_2O$ 10,000 ppm (as $CaCO_3$); $NaHCO_3$ 20,000 ppm (as $CaCO_3$); PBTC 2,000 ppm (as PBTC).

### Instrumentation

A DR-2000 Spectrophotometer from the Hach Co. (Loveland, Co, U.S.A.) was used for halogen and phosphate analyses. Protocols were followed according to the literature /14/. $Ca^{2+}$ was measured by Atomic Absorption Spectroscopy.

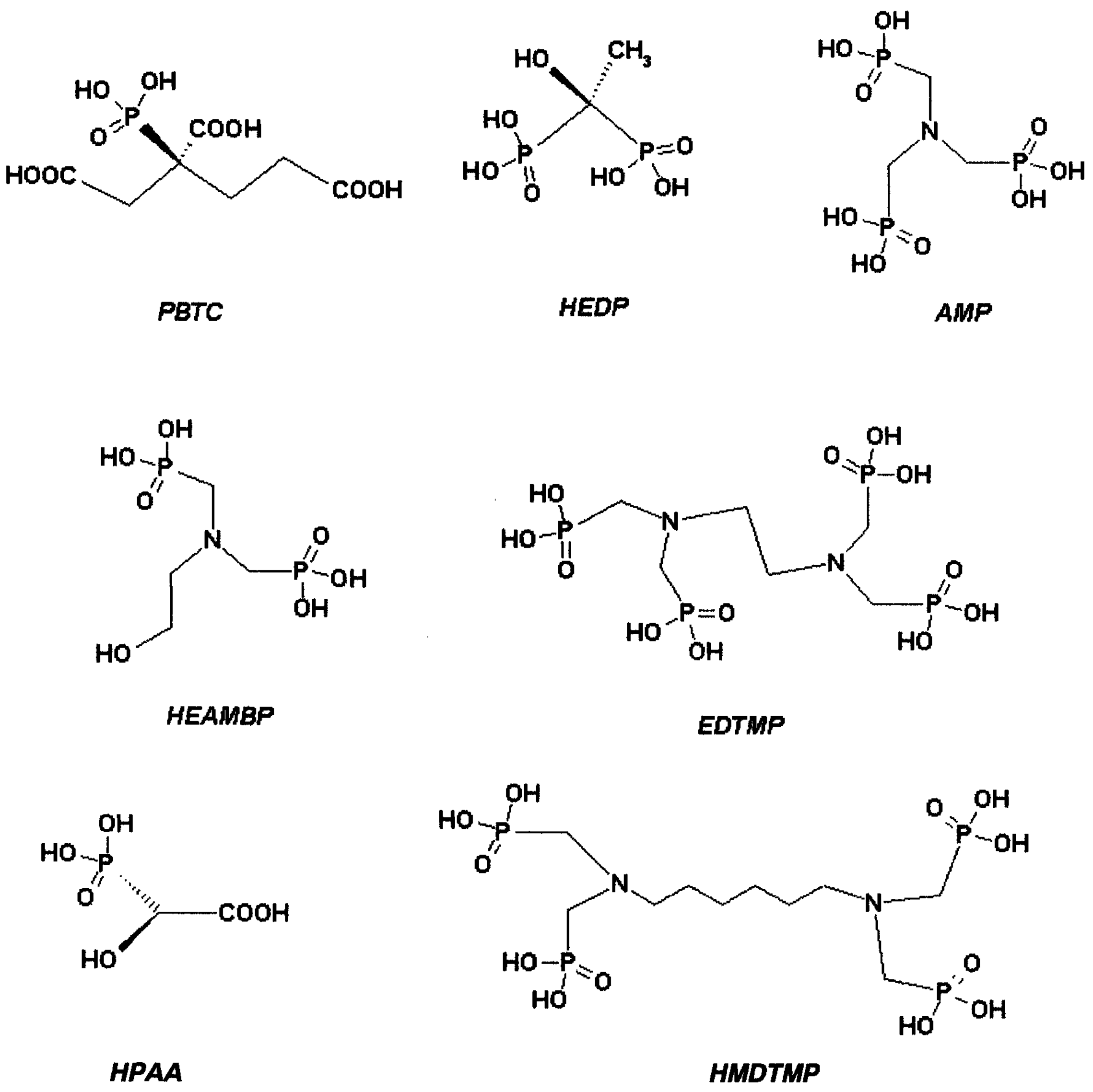

**Fig. 1:** Representative schematic structures of some representative mineral scale inhibitors. The symbol abbreviations are as follows: AMP amino-*tris*-methylene phosphonate, HEDP 1-hydroxyethylidene-1,1-diphosphonic acid, PBTC 2-phosphonobutane-1,2,4-tricarboxylic acid, HMDTMP hexamethylene-N,N',N',N'-diamine tetramethylenephosphonic acid, EDTMP ethylene-N,N'-diamine tetramethylenephosphonic acid, HPAA hydroxyphosphonoacetic acid.

### Calcium Tolerance Procedure

An amount of the $Ca^{2+}$ stock solution (400 mL) was placed in a glass container, which in turn was placed in a water bath and allowed to achieve the desired temperature of 54 °C (monitored by a thermosensor, built into the pH probe) under continuous stirring (300 rpm). The solution pH was kept at 9.00 ± 0.05 units using a pH controller coupled with a syringe pump supplying 0.1 N NaOH (set at 0.50 mL/min). NaOH addition was used for pH adjustment purposes only. Upon temperature equilibration, the PBTC solution was continuously fed into the test solution at 0.50 mL/min rate, using a different syringe pump. During PBTC addition solution pH was kept at 9.00 ± 0.05 units. Solution turbidity (at 420 nm), conductivity and pH were monitored. Signals were stored in a computer at 1 data point/min. At a certain critical PBTC concentration the slope of transmittance started to decrease, indicating turbidity increase due precipitate formation. Calcium tolerance was calculated based on the amount of PBTC (in ppm) at the onset of precipitation. Calculations also took into account dilution effects from external addition of solutions. The same procedure was followed for HEDP and AMP.

### $CaCO_3$ Scale Inhibition Test

$Ca^{2+}$, $Mg^{2+}$, and $HCO_3^-$ are expressed as ppm $CaCO_3$, whereas PBTC as ppm actives. Appropriate amounts of stock solutions were used to achieve final concentrations of $Ca^{2+}$ (800 ppm), $Mg^{2+}$ (200 ppm), and $HCO_3^-$ (800 ppm). In a volumetric flask $Ca^{2+}$, $Mg^{2+}$ were mixed and then the appropriate amount of PBTC was added. Finally, the desired amount of $NaHCO_3$ was added and the remaining volume was made up with de-ionized water. The final volume of the test solution was 100 mL. The solution was then transferred to an Erlenmeyer flask. The flask was covered and placed in a water bath maintained at 43 °C. The solution inside the flask was under constant stirring with a magnetic stirring bar. pH 9.0 was maintained by addition 0.1 N NaOH *via* an auto-titrator. After a time period of 2 h the flask was removed from the water bath and a sample was filtered through a 0.45 μ filter. Analysis by atomic absorption spectroscopy gave the concentration of soluble $Ca^{2+}$. The remaining solution was covered and stored *unstirred* at room temperature. A second set of samples was withdrawn from just below the surface 24 h after the pH was first raised to 9.0. The analytical results of these *unfiltered* samples yielded the dispersed $Ca^{2+}$ concentration. No further pH adjustment was made before the 24 h measurement.

It should be noted that the experimental water used in the present study cannot cover the whole range and variability of natural waters used for cooling purposes. It represents, however, a good "model" for the precipitation chemistry taking place in most process cooling waters. Although other metal ions can form foulant salts in supersaturated cooling waters they were not included in the study because they represent a minority of problems. Such sparingly soluble electrolytes will be subjects of future reports.

### Biocide Resistance Procedure

Appropriate amounts of stock solutions were used to achieve final concentrations of $Ca^{2+}$ (200 ppm), $Mg^{2+}$ (100 ppm), and $HCO_3^-$ (200 ppm). In five different volumetric flasks $Ca^{2+}$, $Mg^{2+}$ were mixed and then the appropriate amount of PBTC was added to give 5 ppm actives. Each biocide was added to the appropriate flask at a 5 ppm dosage (as total $Cl_2$). Solution temperature was 25 °C. pH was adjusted and maintained at 8.3. An aliquot was withdrawn after 1 h and was analyzed for phosphate (o-$PO_4^{3-}$) by the molybdophosphoric method /14/.

### RESULTS AND DISCUSSION

### Inhibitory Effect of PBTC on $CaCO_3$ Crystal Growth and Crystal Modification

Inhibitors are often required to control scale formation under very stressful conditions of metal ion and carbonate concentration and pH. Instrument malfunction occasionally results into increase of cycles of concentration well beyond the specification of the scale inhibition program used. pH sensors left uncalibrated for long periods often give erroneous measurements. As a result, operating conditions of high supersaturation levels and high pH are not unusual. Scale inhibitors can protect the system from such operational upsets and, consequently, unwanted deposits.

In the present study high hardness conditions were used to model a situation where there is uncontrollable increase of the cycles of concentration. This could very well occur when a malfunctioning conductivity meter does not properly allow supersaturated water to escape from the system (blowdown) and be replaced with make-up (fresh) water.

$CaCO_3$ is the only insoluble salt precipitating under the conditions studied. Although $Mg^{2+}$ ions are added in the system (to "model" realistic process cooling waters) they do not cause precipitation of $Mg(OH)_2$, which forms at pH regions above 10. Precipitation of $MgCO_3$ is not a possibility, since its solubility is much higher than that of $CaCO_3$.

Under the experimental conditions studied, PBTC can inhibit the formation of $CaCO_3$ up to 350 ppm at a 60 ppm dosage level (Table 1, Figure 2). Its performance is comparable to that of HEDP and AMP under similar conditions /12a/. Its effectiveness shows an upward trend as the actives level increases, Figure 2.

Under the conditions studied, PBTC does not appear to offer any dispersancy properties. This is concluded based on the results obtained for dispersed Ca (Table 1, right column). Lower numbers for dispersed Ca indicate that $CaCO_3$ formation is continued over 24 h and that the $CaCO_3$ formed cannot remain suspended close to the air-water interface, where the sampling point is. In the presence of 15 or 30 ppm PBTC, 20 ppm of $Ca^{2+}$ are lost to $CaCO_3$. At the level of 60 ppm PBTC, 40 ppm of $Ca^{2+}$ precipitate into $CaCO_3$. Although these results do not constitute a complete kinetics study they are initial indications of the inhibitory activity of PBTC against $CaCO_3$ crystal formation under these "harsh" hardness conditions. Suitable kinetics studies will be performed in our laboratories.

It is interesting to note that scale inhibitor performance largely depends on inhibitor structure. For example, hydroxy-ethylidenediphosphonate (HEDP) exhibits excellent scale inhibition properties for $CaCO_3$, whereas methylenediphosphonate has virtually no activity (unpublished results). PBTC is one of the most effective scale inhibitors. Citrate, a close structural analog to PBTC but with no phosphonate group, shows little activity /15/.

Phosphonates in general also act as crystal modifiers. The precipitated scale crystals often exhibit

**Table 1**

$CaCO_3$ Crystal Growth Inhibition Results in the Presence of PBTC.[a]

| Experiment | PBTC (ppm as actives) | Soluble $Ca^{2+}$ (ppm as $CaCO_3$) | Dispersed $Ca^{2+}$ (ppm as $CaCO_3$) |
|---|---|---|---|
| 0 | 0 | 5 | 3 |
| 1 | 15 | 280 | 260 |
| 2 | 30 | 310 | 290 |
| 3 | 60 | 350 | 310 |

[a] Conditions: pH 9.0, $Ca^{2+}$ 800 ppm (as $CaCO_3$), $Mg^{2+}$ 200 ppm (as $CaCO_3$), $HCO_3^-$ 800 ppm (as $CaCO_3$), T = 43 °C.

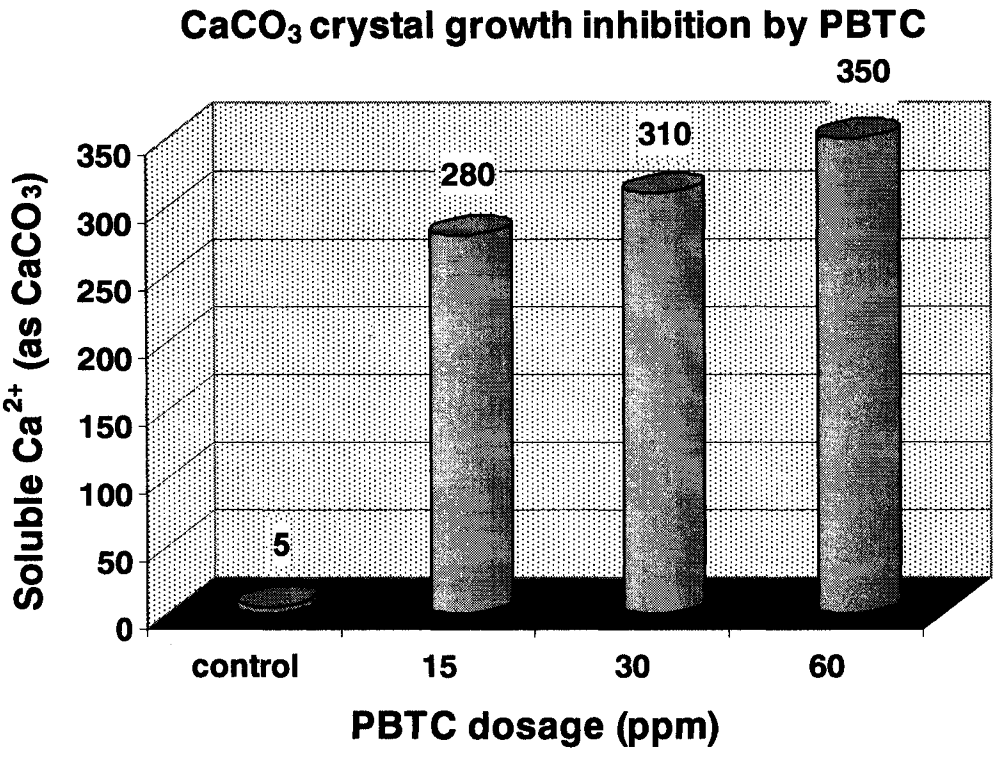

**Fig. 2:** Inhibitory effect of PBTC on $CaCO_3$ crystal growth based on soluble $Ca^{2+}$.

distortions caused by preferential phosphonate adsorption onto specific crystallographic planes of the salt crystal. We isolated $CaCO_3$ crystals precipitated without additives and those that crystallized from solutions containing PBTC. The SEM images are shown in Figure 3. Untreated $CaCO_3$ crystallizes as prismatic calcite (one of the three $CaCO_3$ polymorphs, the other two being vaterite and aragonite) with well-defined edges. The presence of PBTC causes obvious changes to $CaCO_3$ crystal morphology with rounded corners and formation of aggregates. However one should also notice that the crystals formed in the presence of PBTC are statistically fewer in number. Aggregation of $CaCO_3$ crystals can be noticed in the presence of certain additives. FT-IR of PBTC-treated $CaCO_3$ crystals showed presence of bands associated with the $-PO_3^{2-}$ group (results not shown). This can be interpreted by inhibitor incorporation either within the $CaCO_3$ lattice, or, more likely, at the edges of $CaCO_3$ crystals.

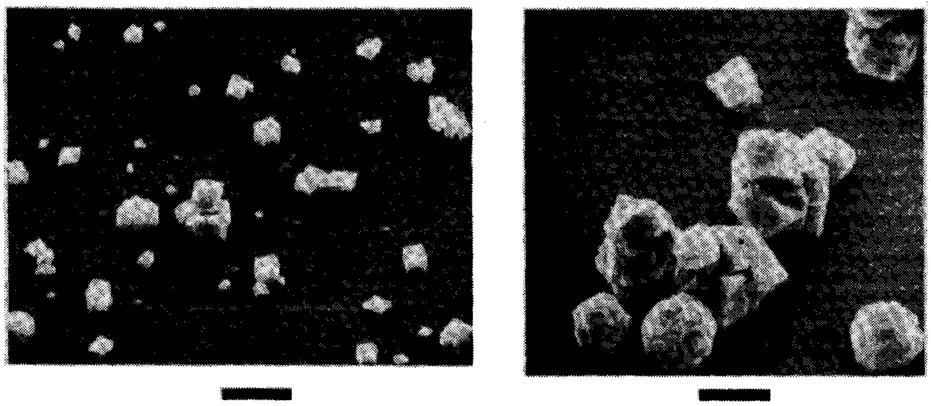

**Fig. 3:** Influence of PBTC on crystal morphology of $CaCO_3$. Crystallization of without additives (left, bar = 20 μ), and with 15 ppm PBTC (right, bar = 10 μ).

### Resistance to oxidizing biocides

Certain scale inhibitors, such as HEDP, AMP and other aminomethylene phosphonates, have well-known susceptibility to oxidation by chlorine or bromine-based biocides (necessary to control microbiological growth) /16/. Orthophosphate ($PO_4^{3-}$), one of the degradation products, can cause calcium phosphate scale deposition.

PBTC is virtually immune to oxidizing biocides and does not decompose to any appreciable extent, at least at "normal" biocide dosage (see Table 2 and Figure 4). Biocide level used in these experiments (5 ppm) is considered to be fairly high (levels well below 1 ppm are considered common. Even under these "high stress" conditions PBTC does not show any appreciable decomposition.

For comparison, we are presenting data taken from the literature on the effects of various biocides on PBTC. These are taken from the work of Vaska /3a/ and are presented in Table 3 and plotted in Figure 5. It is evident that Vaska's results indicate a more pronounced decomposition of PBTC. This occurs because of the following reasons: (a) The reaction time is longer (2 h *vs.* 1 h in our study) than our experiments (b) The

**Table 2**

Effect of various oxidizing biocides and their stabilized analogs on PBTC.[a]

| Experiment | PBTC (ppm as actives) | Biocide, dosage | % reversion to $PO_4^{3-}$ |
|---|---|---|---|
| 4 | 5 | Control (no biocide) | 2 |
| 5 | 5 | $ClO^-$ (5 ppm as $Cl_2$) | 3 |
| 6 | 5 | $BrO^-$ (5 ppm as $Br_2$) | 5 |
| 7 | 5 | Stabilized Bromine (5 ppm as $Br_2$) | 4 |
| 8 | 5 | Stabilized Chlorine (5 ppm as $Cl_2$) | 2 |

[a] Conditions: sample tested after 1 h, T = 25 °C, pH 8.3

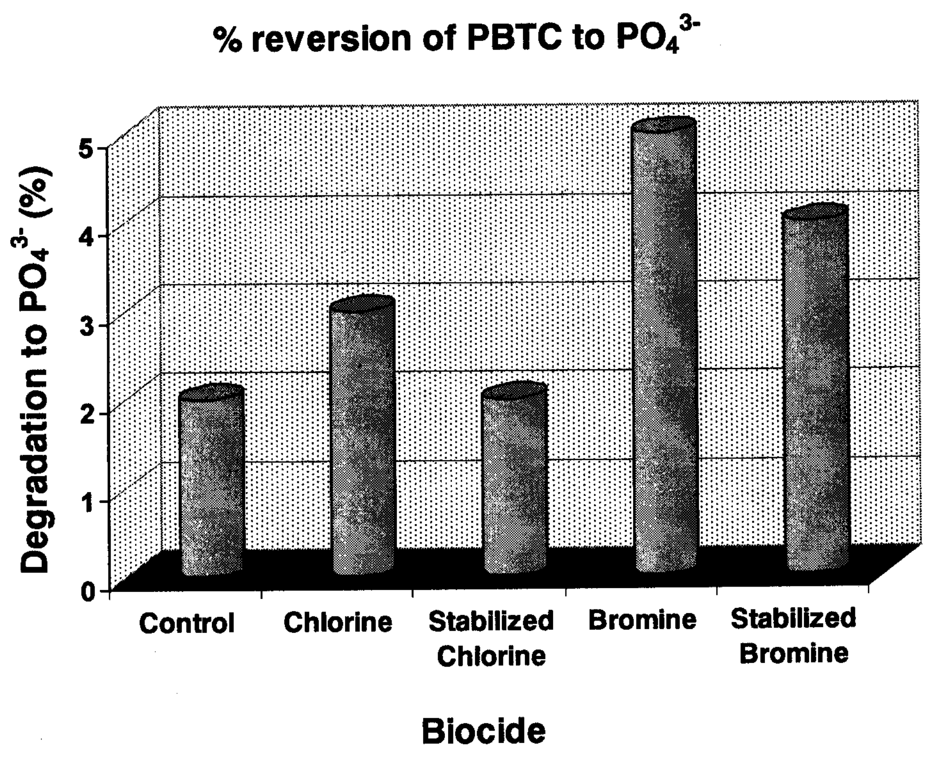

**Fig. 4:** Reversion of PBTC to phosphate (o-$PO_4^{3-}$) in the presence of chlorine and bromine, and their stabilized analogs (see definition of terms in Glossary).

**Table 3**

Effect of various biocides on PBTC.[a]

| Experiment | PBTC (ppm) | Biocide, dosage | % reversion to $PO_4^{3-}$ |
|---|---|---|---|
| 9 | 19 | $ClO^-$ (7 ppm as $Cl_2$) | 25 |
| 10 | 20 | $BrO^-$ (16.6 ppm as $Br_2$) | 5 |
| 11 | 21 | BCDMH[b] (21 ppm as BCDMH) | 10 |
| 12 | 19 | $ClO_2$ (14 ppm as $ClO_2$) | 20 |

[a] Conditions: DI water, pH 8.0, T = 70 °C, 2h sampling. Data were taken from reference 3a

[b] BCDMH = 1-bromo-3-chloro-5,5-dimethyl-hydantoin

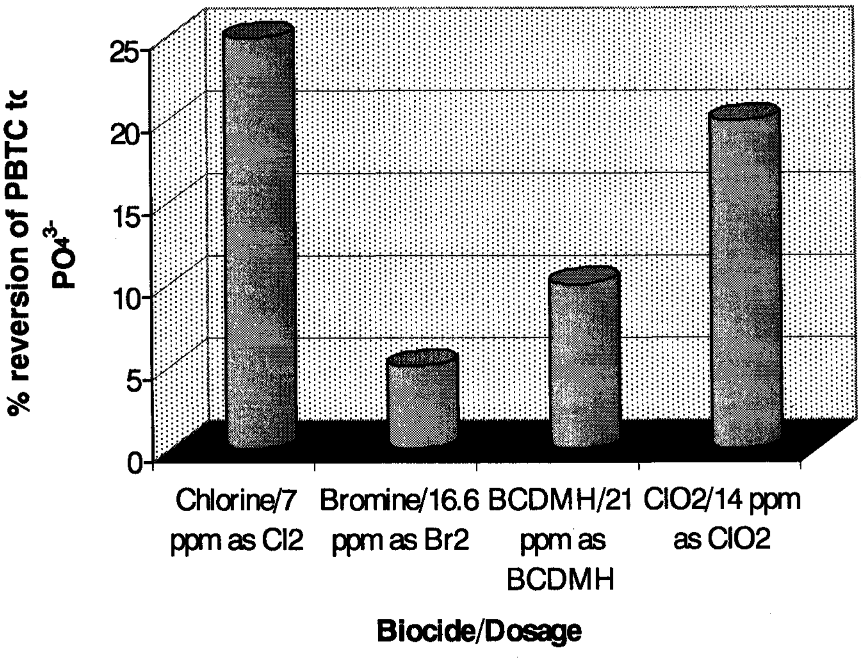

**Fig. 5:** Effect of chlorine, bromine, BCDMH and $ClO_2$ on PBTC degradation (see definition of terms in Glossary).

reaction temperature is much higher (70 °C *vs.* 25 °C in our study) (c) Biocide levels are significantly higher (see Table 3). These are not realistic for common cooling water biocide applications. (d) The reaction pH is somewhat lower (8.0 *vs.* 8.3 in our study). This affects effectiveness of hypochlorite, which functions more effectively in lower pH regions.

### Calcium tolerance

Certain applications of organophosphonate scale inhibitors are based on their precipitation as insoluble species with ions such as $Ca^{2+}$, $Sr^{2+}$, $Ba^{2+}$, *etc.* In geothermal wells, for example, precipitation of scale inhibitors as alkaline earth salts is desirable. Large amounts of inhibitor are "squeezed" in the oilfield well and remain there for a specified amount of time, during which the inhibitor precipitates with alkaline earth metals found in the high-salinity brine and eventually deposits onto the rock formation. Once the well is opened again for operation the metal-inhibitor salts slowly dissolve to provide adequate levels of scale inhibitor in solution /17/. Controlled dissolution of these salts is essential, as fast dissolution will lead to chemical wastage and slower dissolution will result in inefficient scale control.

In cooling water applications (particularly in open recirculating sustems) adequate levels of inhibitor at all times is essential. In these systems, occasionally high supersaturation levels of $Ca^{2+}$, coupled with inhibitor overfeeding may lead to precipitation of insoluble Ca-inhibitor precipitates. Such precipitates can be detrimental to the entire cooling water treatment program for several reasons:

(a) They cause depletion of soluble inhibitor, and, subsequently, poor scale control because there is little or no inhibitor available in solution to inhibit scale formation.

(b) They can act as potential nucleation sites for other scales.

(c) They can deposit onto heat transfer surfaces (they usually have inverse solubility properties) and cause poor heat flux, much like other known scales, such as calcium carbonate, calcium phosphate, etc.).

(d) If the phosphonate inhibitor in the treatment program has the purpose of corrosion inhibition, its precipitation as a Ca salt will eventually lead to poor corrosion control.

Based on the above, resistance to precipitation is a useful property of a scale inhibitor. Calcium tolerance is defined as the ability of a certain chemical compound to remain soluble in the presence of calcium ions. It usually decreases as pH increases. This is because at higher pH's the degree of deprotonation of inhibitors (usually phosphonates or carboxylates) is higher.

Calcium tolerance becomes very critical as cycles of concentration increase. An efficient inhibitor must interact strongly with $Ca^{2+}$, but must be sufficiently soluble to remain in the system. Ca-inhibitor salt precipitation is well known for phosphonate as well as carboxylate-based inhibitors /18/. Three of the most commonly used scale inhibitors, AMP, PBTC, and HEDP, were studied comparatively with respect to their Ca tolerance. The results are found in Table 4 and in Figure 6. The above inhibitors can be rated according to their Ca tolerance in descending order (as ppm soluble inhibitor per 1000 ppm of $Ca^{2+}$ as $CaCO_3$): PBTC (185 ppm) > AMP (12 ppm) > HEDP (8 ppm). The above results indicate that use of PBTC in applications where maintenance of sufficient inhibitor levels is critical should be preferred. On the other hand, in other applications where precipitation of the scale inhibitor is desirable, PBTC is not the best choice, due to its unwillingness to form precipitates with metal ions.

A Ca-PBTC complex does not form under the conditions used in the $CaCO_3$ Scale Inhibition Test because of its high solubility.

**Table 4**

Comparative Calcium tolerance of HEDP, AMP, and PBTC.

| **Phosphonate** | **$Ca^{2+}$ level (ppm as $CaCO_3$)** | **Phosphonate level[a] (ppm actives)** |
|---|---|---|
| HEDP | 1000 | 8 |
| AMP | 1000 | 12 |
| PBTC | 1000 | 185 |

[a] At the time of Ca-inhibitor salt precipitation.

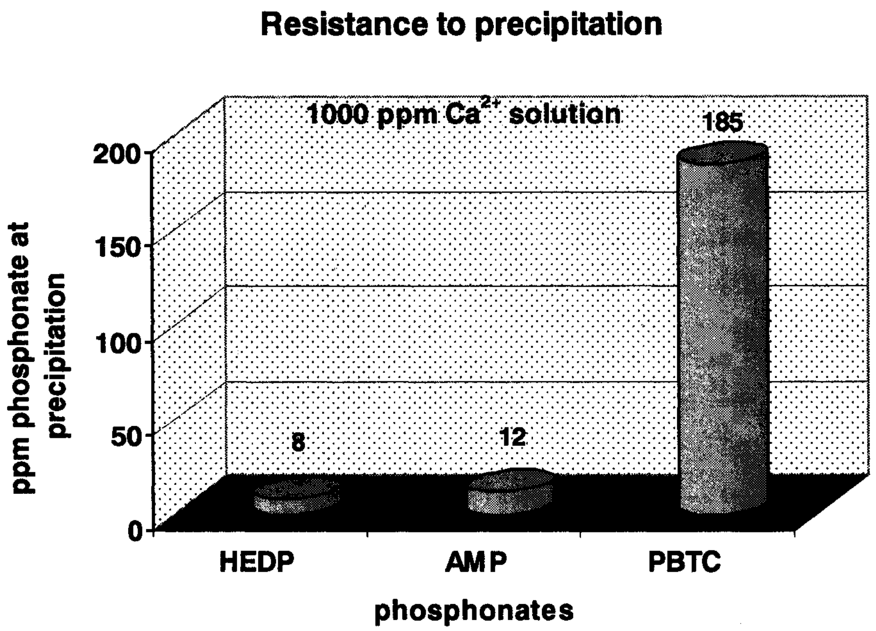

**Fig. 6:** Comparison of precipitation resistance with $Ca^{2+}$ of HEDP, AMP, and PBTC.

### CONCLUSIONS

The definition of the ideal scale inhibitor varies according to the particular application. The scale inhibitor operating in open recirculating cooling water applications must possess desirable properties such as: (a) excellent scale inhibition performance, (b) high metal ion tolerance (resistance of metal-inhibitor salt to precipitate out of solution), (c) stability towards oxidizing biocides, (d) thermal stability (for high temperature applications), and (d) low production cost.

The results of the present study are summarized as follows:

(a) PBTC is an effective $CaCO_3$ inhibitor under conditions of high hardness and alkalinity.

(b) It is essentially immune to decomposition by oxidizing biocides at normal biocide levels. This makes PBTC the ideal choice in water systems treated with oxidizers for microbiological control.

(c) PBTC possesses high calcium tolerance and its Ca complex salt does not precipitate under conditions of extreme hardness.

The systematic study of a plethora of phosphonates and mixed phosphonates/carboxylates and their inhibitory activity against metal salt scalants under conditions representing various industrial water treatment applications are currently underway in our research efforts /19/.

### ACKNOWLEDGMENTS

The help of Dr. Dimitri Kusnetsof with the calcium tolerance experiments is gratefully acknowledged.

### REFERENCES

1. *Calcium Carbonate*: (a) *J. Chem. Soc. Faraday Trans.* **1984**, *80*, 1181. (b) Xyla, G.A.; Giannimaras, E.K.; Koutsoukos, P.G. *Colloids Surf.* **1991**, *53*, 241. (c) Gal, J.-Y.; Bollinger, J.-C.; Tolosa, H.; Gache, N. *Talanta* **1996**, *43*, 1497. (d) Van Der Weijden et al. *J. Cryst. Growth* **1997**, *171*, 190. (e) Gomez-Morales, J. et al. *J. Cryst. Growth* **1996**, *166*, 1020. (f) Zafiropoulou, A.; Dalas, E. *J. Cryst. Growth* **2000**, *219*, 477. (g) Dalas, E. *J. Cryst. Growth* **2001**, *222*, 287.
2. *Calcium Phosphates*: (a) Z. Amjad "Calcium Phosphates in Biological and Industrial Systems" Kluwer Academic Publishers: Boston 1998. (b) Koutsoukos, P.; Amjad, Z.; Tomson, M.B.; Nancollas, G.H. *J. Am. Chem. Soc.* **1980**, *102*, 1553. (c) Sørensen, J.S.; Lundager Madsen, H.E. *J. Cryst. Growth* **2000**, *216*, 399. (d) Golubev, S.V.; Pokrovsky, O.S.; Savenko, V.S. *J. Cryst. Growth* **1999**, *205*, 354. (e) Deluchat, V.; Bollinger, J.-C.; Serpaud, B.; Caullet, C. *Talanta* **1997**, *44*, 897. (f) F. H. Browning, H.S. Fogler, *SPE Production & Facilities* **1995**, *August*, 144. (g) Amjad, Z. *Tenside Surf. Det.* **1997**, *34*, 102. (h) US patent 5,346,010. (i) Zieba, A.; Sethuraman, G.; Perez, F.; Nancollas, G.H.; Cameron, D. *Langmuir* **1996**, *12*, 2853.

3. (a) Vaska, M. *Industrial Water Treatment* **1993**, *March/April*, p. 39. (b) Johnson, D.A.; Fulks, K.E.; Meier, D.A. Corrosion/86, Paper No. 403, National Association of Corrosion Engineers, Houston, TX, 1986.
4. (a) S. Zaheer Akhtar, *Power Engineering* **2000**, *October*, 63. (b) J. Katzel, *Plant Engineering* **1989**, *27 (April)*, 32. (c) Power Special Report, *Power* **1973**, *March*, S-1. (d) E.C. Elliot, *Power* **1985**, *December*, S-1. (e) R. Burger, *American Power Conference* **1995**, *Vol. 57*, p. 1363. (f) R. Burger, *American Power Conference* **1994**, *Vol. 56*, p. 1085.
5. (a) Demadis, K.D. *Chemical Processing* **2003**, *May*, p. 29. (b) Young, P.R.; Stuart, C.M.; Eastin, P.M. *Cooling Technology Institute 1993 Annual Meeting, Technical Paper # TP93-11.* (c) Dubin, L. US Patents 4,532,047 and 4,584, 104. (d) Meier, D.A.; Dubin, L. Paper No. 334, National Association of Corrosion Engineers, Houston, TX, 1987. (e) Gill, J.S. *Materials Performance* **1998**, *November, p. 41.* (f) Dubin, L.; Dammeier, R.L.; Hart, R.A. *Materials Performance* **1985**, *October, p. 27.* (g) Young, P.R. Paper No. 466, National Association of Corrosion Engineers, Houston, TX, 1993.
6. (a) *Mineral Scale Formation and Inhibition,* Amjad, Z. Ed.; Plenum Press: New York, 1995 and references therein. (b) P. Ashdown, D. Ashworth, W. Guthrie, *Performance Chemicals Europe* **1999**, *November/December*, 28. (c) Matty, J.M.; Tomson, M.B. *Appl. Geochem.* **1988**, *3*, 549. (d) Tomson, M.B. *J. Cryst. Growth* **1983**, *62*, 106.
7. (a) Amjad, Z.; Pugh, J.; Zibrida, J.; Zuhl, B. *Materials Performance* **1997**, *January*, p. 32. (b) Rieger, J.; Hadicke, E.; Rau, I.U.; Boeckh, D. *Tenside Surf. Det.* **1997**, *6*, 430. (c) Amjad, Z. *Tenside Surf. Det.* **1997**, *34*, 102. (d) J.E. Hoots, G.A. Crucil, Corrosion/86, Paper No. 13, National Association of Corrosion Engineers, Houston, TX, 1986. (e) E.B. Smyk, J.E. Hoots, K.P. Fivizzani, K.E. Fulks, Corrosion/88, Paper No. 14, National Association of Corrosion Engineers, Houston, TX, 1988. (f) C.C. Pierce, J.E. Hoots in Chemical Aspects of Regulation of Mineralization, C.S. Sikes and A.P. Wheeler Eds., University of Alabama Publication Services, Alabama 1988, p. 53. (g) C.C. Pierce, D.A. Grattan, Corrosion/88, Paper No. 205, National Association of Corrosion Engineers, Houston, TX, 1988.
8. (a) Dequest: Phosphonates by Solutia (2054 Phosphonates for scale and corrosion control, chelation, dispersion), Publication # 7450006A. (b) Dequest: Phosphonates by Solutia (2060-S, 2066 & 2066-A Phosphonates: metal ion control agents), Publication # 7459369. (c) Dequest: Phosphonates by Solutia (Introductory Guide), Publication # 7459151B. (d) Dequest: Phosphonates by Solutia (2000 & 2006 Phosphonates for scale and corrosion control, chelation, dispersion), Publication # 7459023B. (e) Silvestre, J.-P.; Dao, N. Q.; Leroux, Y. *Heteroatom Chemistry*, **2001**, *12*, 73, supplement to the Special Issue on the chemistry and biochemistry of bisphosphonates and aminophosphonates, **2000**, *Vol. 11*, Issue 7. (f) Silvestre, J.-P.; N. Q. Dao, N. Q.; Salvini, P. *Phosphorus, Sulfur and Silicon* **2002**, *177*, 771.
9. (a) Bosbach, D.; Hochella, M.F. *Chemical Geology* **1996**, *132*, 277. (b) Amjad, Z. *Langmuir* **1991**, *7*, 600. (c) Fulks, K.E.; Yeoman, A.M. Corrosion/83, Paper No. 279, National Association of Corrosion Engineers, Houston, TX, 1983. (d) Hale, E.R.; Hoots, J.E.; Nicolich, S.N. *Power Engineering* **1999**, *September*, 21. (e) Amjad, Z.; *Can. J. Chem.* **1988**, *66*, 2180.
10. (a) Sweeney, F.M.; Cooper, S.D. *Society of Petroleum Engineers International Symposium on Oilfield Chemistry, New Orleans, LA March 2-5, 1993, paper SPE 25159.* (b) Oddo, J.E.; Tomson, M.B. Corrosion/92, Paper No. 34, National Association of Corrosion Engineers, Houston, TX, 1992. (c)

Xiao, J.; Kan, A.T.; Tomson, M.B. *American Chemical Society-Division of Fuel Chemistry, Symposium Preprints* **1998**, *43*, 246. (d) Oddo, J.E.; Tomson, M.B. *SPE Production & Facilities* **1994**, *February*, 47.

11. (a) Darling, D.; Rakshpal, R. *Materials Performance* **1998**, *December*, 42. (b) Strauss, S.D. *Power* **1992**, *September*, 17. (c) Farooqi, I.H.; Nasir, M.A.; Quraishi, M. *Corr. Prev. & Control* **1997**, *October*, 129. (c) Mosayebi B.; Kazemeini M.; Badakhshan A. *Br. Corr. J.* 2002, 37, 217-224.
12. (a) Demadis, K.D.; Yang, B.; Young, P.R.; Kouznetsov, D.L.; Kelley, D.G. in *Advances in Crystal Growth Inhibition Technologies*; Amjad, Z., Editor; Plenum Press, New York: **2000**, Chapter 16, p. 215. (b) Sallis, J. D. In *Calcium Phosphates in Biological and Industrial Systems*; Amjad, Z., Ed.; Kluwer Academic Publishers: New York, 1998; Chapter 8, p 173. (c) Amjad Z. in Amjad Z, Ed. *Mineral Scale Formation And Inhibition*, New York: Plenum Press, 1995. p. 207. (d) Dubin, L. Corrosion/80, Paper No. 222, National Association of Corrosion Engineers, Houston, TX, 1980. (e) Ashdown, P.; Ashworth, D.; Guthrie, W. *Performance Chemicals Europe* **1999**, *November/December*, 28.
13. (a) Demadis, K.D. in *Compact Heat Exchangers and Enhancement Technology for the Process Industries*, R.K. Shah, Editor, Begell House Inc., New York, 2003, p. 483. (b) Oddo, J.E.; Tomson, M.B. Paper No. 34, National Association of Corrosion Engineers, Houston, TX, 1992. (c) Sarig, S.; Ginio, O. *J. Phys. Chem.* **1976**, *80*, 256. (d) Oddo, J.E.; Tomson, M.B. *SPE Production & Facilities* **1994**, *February*, 47. (e) Cowan, J.C.; Weintritt, D.J. *Water-Formed Scale Deposits*, Gulf Publishing Co. Houston, TX, 1976, p. 93.
14. (a) *Standard Methods for the Examination of Water and Wastewater*, American Public Health Association, Washington D.C., 19th Edition, 1995. (b) *Water Analysis Handbook*, The Hach Company, Loveland, Colorado, U.S.A.
15. (a) Grases, F.; Millan, A.; Garcia-Raso, A. *J. Cryst. Growth* **1988**, *89*, 496. (b) Vdovic, N.; Kralj, D. *Coll. Surf. A: Phys. Eng. Aspects* **2000**, *161*, 499.
16. L. Scerbo, paper 471, National Association of Corrosion Engineers, Houston, TX, 1993.
17. (a) Browning, F.H.; Fogler, H.S. *AIChE Journal* **1996**, *42*, 2883.
18. (a) Amjad, Z.; Zuhl, R.W.; Zibrida, J.F., Association of Water Technologies 2003 Annual Convention, September 17 – 20, 2003, Phoenix, AZ. (b) Wohlever, J.A.; Amjad, Z.; Zuhl, R.W. in Advances in Crystal Growth Inhibition Technologies, Amjad, Z. Editor, Kluwer Academic Publishers, New York, NY (2001). (c) Masler, W.F. Amjad, Z. Corrosion/88, Paper No. 11, National Association of Corrosion Engineers, Houston, TX, 1988.
19. Demadis, K.D.; Katarachia, S. *Phosphorus Sulfur Silicon* **2004**, *179*, 627.

